# Epidemiological impacts and cost-effectiveness of daily and on-demand oral pre-exposure prophylaxis among key HIV populations in China: An economic evaluation

**DOI:** 10.1017/S095026882510068X

**Published:** 2025-12-19

**Authors:** Shu Su, Yuhang Wei, Wei Dong, Jiajun Sun, Yuxuan Li, Wendi Zhang, Qingxian Song, Zunyou Wu, Rui Zhao, Lei Zhang

**Affiliations:** 1 Department of Epidemiology and Biostatistics, The Second Affiliated Hospital of Chongqing Medical University, Chongqing, China; 2 China-Australia Joint Research Center for Infectious Diseases, School of Public Health, Xi’an Jiaotong University Health Science Center, Xi’an, Shaanxi, China; 3 National Center for AIDS/STD Control and Prevention, Chinese Center for Disease Control and Prevention, Beijing, China; 4 Artificial Intelligence and Modelling in Epidemiology Program, Melbourne Sexual Health Centre, Alfred Health, Melbourne, Australia; 5 Shenzhen Hospital of Southern Medical University, Shenzhen, China; 6 Phase I clinical trial research ward, The Second Affiliated Hospital of Xi’an Jiaotong University, Xi’an, Shaanxi, China; 7 School of Translational Medicine, Faculty of Medicine, Nursing and Health Sciences, Monash University, Melbourne, Australia

**Keywords:** China HIV elimination, cost-effectiveness analysis, daily PrEP, key populations, on-demand PrEP, pre-exposure prophylaxis

## Abstract

This study assessed the impact and cost-effectiveness of pre-exposure prophylaxis (PrEP) in reducing HIV infections and HIV-related deaths among four key populations in China: men who have sex with men (MSM). Female sex workers (FSW), people who inject drugs (PWID), and HIV-negative partners of serodiscordant couples (SDC). Decision-analytic Markov models simulated HIV transmission and progression in cohorts of 100,000 adults over 40 years under three strategies: no PrEP, daily oral PrEP, and on-demand oral PrEP evaluated nationaly and high-incidence provinces. Cost-effectiveness was measured using a willingness-to-pay threshold of US$37,653 per QALY. Across all populations, on-demand PrEP was the most cost-effective strategy. Among MSM, it was cost-effective both nationwide (ICER: $4,554/QALY) and in high-incidence provinces (ICER: $1,045-2,129/QALY), reducing new infections by 24.7%. Daily PrEP was also const-effective for MSM nationally and prevented 19.9% of infections. For FSW, on-demand PrEP was cost-effective in high-incidence provinces (ICER: $25,399-37,045/QALY), reducing infections by 21.8%-22.5%. For PWID, it was cost-effective in high-incidence provinces (ICER: $10,361-29,560/QALY), reducing infections by 15.5%-17.9%. For HIV-negative partners of SDC, on-demand PrEP was cost-effective both nationally and in high-incidence provinces, reducing infections by 24.0%. Overall, on-demand PrEP offers substantial health and economic benefits, particularly for HIV-negative partners of SDC and high-incidence regions.

## Introduction

The global HIV epidemic remains severe [1], with key populations and their sexual partners accounting for 70% of all HIV cases worldwide in 2022 [2]. Sexual transmission is the predominant route, particularly in China, where 97% of HIV cases are attributed to sexual contact [3]. Therefore, preventing HIV transmission within key populations (including men who have sex with men (MSM), female sex workers (FSW), people who inject drugs (PWID), and the HIV-negative partners of serodiscordant couples (SDC) would contribute to achieve the goal of ending HIV by 2030 [4].

Pre-exposure prophylaxis (PrEP) has emerged as a highly effective HIV prevention strategy involving the administration of specific antiretroviral drugs to individuals who are HIV negative but at risk of infection [5]. Its efficacy in HIV prevention has been substantiated by multinational, randomized, placebo-controlled clinical trials conducted on MSM, heterosexual men, and women. These trials indicated that consistent adherence to PrEP provides over 90% protection against HIV [6]. To maximize its effectiveness, PrEP is available in two different regimens: daily and on-demand, allowing individuals to choose the most suitable option based on their lifestyle and risk level. Daily and on-demand PrEP differ in dosing frequency and timing, catering to individual preferences and risk profiles [7]. Daily PrEP involves taking antiretroviral drugs at regular intervals, suitable for individuals with frequent or unpredictable sexual activity [8, 9]. On the other hand, on-demand PrEP, also known as event-driven PrEP or 2-1-1 dosing, involves taking a double dose 2–24 h before exposure, followed by one dose per day until at least 48 h after the last exposure [9]. It is intended for those with infrequent sexual activity or anticipating specific high-risk scenarios [10].

Despite PrEP’s proven effectiveness in preventing HIV acquisition, its costs remains a concern, particularly in a densely populated developing country like China [11–15]. Higher population density allows for centralized resource allocation, reducing per capita costs and improving accessibility. While both daily and on-demand PrEP have been available in China since 2020 [16], they have not yet been integrated into a nationwide programme or even regional implementation for these key populations [17]. Previous studies have indicated that social stigma, unequal distribution of healthcare resources, and individual perceptions of HIV risk significantly influence PrEP preference and usage [18, 19]. Moreover, Chinese MSM prefers PrEP to be provided free of charge, regardless of whether it is daily or on-demand PrEP [18], aligning with global findings that the willingness to use PrEP declines as cost increases [20]. Currently, in China, PrEP services are primarily targeted towards MSM. However, access remains limited due to high out-of-pocket costs, as PrEP is not currently covered by public health insurance [21, 22]. The other key populations may face greater stigma and have less access to healthcare, resulting in lower willingness to use PrEP [13, 23, 24].

Existing cost-effectiveness analyses in China have primarily focused on a single key population, most often MSM. For example, Zhang et al. [11] and Jin et al. [25] have demonstrated that both daily and on-demand PrEP can be cost-effective in MSM, providing limited insight into other key groups. More recently, Wu et al. [26] evaluated a combined screening and PrEP strategy among HIV-negative partners in SDC in Zhejiang Province, also finding favourable cost-effectiveness outcomes. Nonetheless, the overall body of evidence remains narrowly focused, with few studies systematically examining multiple key populations within the same analytic framework. Evaluating multiple key populations within a unified framework ensures more efficient resource allocation and facilitates prioritizing interventions based on their relative effectiveness across diverse groups. In contrast, modelling studies in other countries have assessed the cost-effectiveness of PrEP utilization among FSW, PWID, and the HIV-negative partners of SDC [27, 28], but the cost-effectiveness of these approaches in China remains unclear.

Therefore, our study constructed a decision-analytic Markov model to investigate the effectiveness and cost-effectiveness of both daily PrEP and on-demand PrEP strategies for four HIV key populations (MSM, FSW, PWID, and the HIV-negative partners of SDC) in China. The outcomes of this study hold significant implications for the enhancement of HIV prevention and control strategies among key populations and are expected to provide valuable insights into the formulation of HIV PrEP policies in China.

## Methods

### Study design

We conducted a comprehensive economic evaluation using a decision-analytic model to assess the cost-effectiveness of three PrEP strategies: no PrEP, daily PrEP, and on-demand PrEP among four key populations in China (MSM, FSW, PWID, and the HIV-negative partners of SDC) of various HIV incidence regions from a healthcare system perspective. The model was constructed using TreeAge Pro 2020, and the analysis was reported according to the Consolidated Health Economic Evaluation Reporting Standards statement 2022 (details in Supplementary Materials CHEERS 2022) [29].

### Data source

We collected data from a literature search and Chinese and English public databases to parameterise prevalence data, PrEP-related costs, and other model parameters (Table 1). The parameters required for model construction included epidemiological data of key populations and HIV-negative partners of SDC in China, HIV incidence, mortality rates at each stage of disease progression, PrEP effectiveness, and PrEP discontinuation rate. Cost data were collected from the perspective of healthcare providers, including ART (antiretroviral therapy) drugs, adverse drug reaction treatment, PrEP drugs, and monitoring of PrEP (including HIV testing, syphilis testing, gonorrhoea testing, chlamydia testing, and liver and kidney function testing estimated by every 3 months for each strategy). We assumed daily PrEP comes as 30 pills/month, and on-demand PrEP comes as 16 pills/month [41]. Based on the data from Blued, the monthly cost of daily PrEP was estimated at USD $54.25, and on-demand PrEP was USD $28.99. All costs were expressed in 2021 US dollars. The annual transition probabilities and utility scores were derived from published literature on the ART treatment progression and non-ART of HIV infection to AIDS (acquired immunodeficiency syndrome) stage (CD4 < 200) (Table 1).

**Table 1.** Selected input values, ranges, and probability distributions for Chinese HIV key populations

Parameter	Baseline (range)^a^	Distribution	Reference
*PrEP-related costs ($USD)*			
Daily oral PrEP, 30 pills per month	54.25 (±30%)	Gamma (1.24, 0.01)	Blued^b^
On-demand oral PrEP, 16 pills per month	28.99 (±30%)	Gamma (1.24, 0.01)	Blued^b^
*Monitoring on PrEP*			
HIV test (screening), per test	3.55 (±30%)	Gamma (25.00, 6.50)	[11]
HIV test (RNA viral load), per test	232.5 (155.0–310.01)	Gamma (55.25, 0.24)	[25]
HIV test (confirmation), per test	77.5 (29.61–85.19)	Gamma (24.97, 0.28)	[11, 30, 31]
Syphilis test, per test	2.96 (1.49–4.45)	Gamma (24.93, 7.78)	[11, 30]
Gonorrhoea test, 3 sites, per test	22.21 (±30%)	Gamma (24.98, 3.12)	[11]
Chlamydia test, 3 sites, per test	22.21 (±30%)	Gamma (24.98, 3.12)	[11]
Liver and kidney function test, per test	7.75 (±30%)	Gamma (24.95, 3.22)	[25]
Follow-up consultation, per visit	31.0 (±30%)	Gamma (24.98, 0.81)	[25]
*ART cost ($USD)*			
First-line ART drug, annually	646.72(±30%)	Gamma (24.97, 0.04)	[32]
Second-line ART drug, annually	1321.62(±30%)	Gamma (24.97, 0.02)	[32]
First-line ART test, first year, annually	590.09(±30%)	Gamma (24.97, 0.04)	[32]
First-line ART test, subsequent years, annually	260.09(±30%)	Gamma (24.97, 0.10)	[32]
Second-line ART test, first year, annually	701.7(±30%)	Gamma (24.97, 0.04)	[32]
Second-line ART test, sequent year, annually	329.84(±30%)	Gamma (24.97, 0.08)	[32]
Adverse drug reaction CD4 ≥ 200, annually	192.05(±30%)	Gamma (24.97, 0.13)	[33]
Adverse drug reaction CD4 < 200, annually	386.58(±30%)	Gamma (24.97, 0.06)	[33]
Opportunistic infection treatment CD4 < 200, annually	630.9(±30%)	Gamma (24.97, 0.04)	[33]
*Discount rate, annually*	0.03(0–0.05)	Beta (8.14, 272.45)	[34, 35]
*Effectiveness of on-demand PrEP, RR*	0.555(0.353–1)	Beta (150.43, 11.32)	[13]
*Discontinuation of daily PrEP, monthly*	0.334(0.195–0.561)	Beta (5.82, 7.69)	[36]
*Progress of HIV/AIDS no ART, annually*			
200 < CD4 < 350	0.534(0.234–0.888)	Beta (7.36, 6.35)	[37]
350 < CD4 < 500	0.389(0.230–0.606)	Beta (14.21, 22.03)	[37]
CD4 < 500	0.257(0.184–0.353)	Beta (54.62, 157.09)	[37]
*Progress of HIV/AIDS on ART, monthly*			
Stable on CD4 < 200	0.9605(0.5–0.99)	Beta (4.65, 0.32)	[38]
Stable on 200 < CD4 < 350	0.954(0.5–0.99)	Beta (5.09, 0.38)	[38]
Stable on 350 < CD4 < 500	0.9593(0.5–0.99)	Beta (4.73, 0.33)	[38]
*Utility scores*			
No HIV infection	1	–	[39]
Viral suppression	0.935(0.88–0.97)	Beta (143.00, 10.11)	[39]
CD4 ≥ 500 not on ART	0.935(0.88–0.97)	Beta (143.00, 10.11)	[39]
350 ≤ CD4 < 500 not on ART	0.935(0.78–0.97)	Beta (143.00, 10.11)	[39]
200 ≤ CD4 < 350 not on ART	0.818(0.78–0.94)	Beta (132.39, 28.47)	[39]
CD4 < 200 not on ART	0.702(0.7–0.87)	Beta (161.62, 65.69)	[39]
Death	0	…	[39]
*Willingness to pay ($USD)*	37653	…	[40]

a Baseline values, sensitivity analysis ranges, and probability distributions are the same for MSM, FSW, PWID, and the HIV-negative partners of SDC.

b Blued: Currently the largest gay social network app in the world.

### PrEP strategies

Three strategies were modelled based on PrEP use and HIV testing frequency for target populations (MSM, FSW, PWID, and the HIV-negative partners of SDC).

1. No PrEP strategy (status quo): no PrEP use, with background HIV care services (HIV testing every 3 months, access to first- and second-line ART for diagnosed individuals);
2. Daily PrEP strategy: daily oral PrEP (assumed 80% coverage of daily PrEP and discontinuation rate applied), with HIV care services as in no PrEP strategy;
3. On-demand PrEP strategy: on-demand oral PrEP (assumed 80% coverage of on-demand PrEP), with HIV care services as in no PrEP strategy.

We assumed that upon implementation, PrEP coverage would immediately reach the target level (80% in base-case) to reflect an idealized scenario representing the maximum potential impact of widespread PrEP implementation, in line with global prevention targets. To address the variability in real-world uptake, especially among key populations, we further explored alternative coverage scenarios (20% and 50%) in one-way sensitivity analyses. Condom use and its potential protective effect were not included in the model due to limited reliable data across all four key populations. Basic interventions, including ART coverage, were assumed to remain constant across all strategies and were not explicitly implemented. This approach was adopted to isolate and evaluate the incremental impact of introducing PrEP, independent of other background changes in HIV prevention and care. Taking into account individual choice, risk perception, and dynamic behaviours of different key populations, we assumed different discontinuation rates for the daily PrEP of each population, as the discontinuation would affect the effectiveness of the daily PrEP [36].

### Model construction

The study constructed a Markov model to simulate disease progression among key populations susceptible to HIV infection using three PrEP strategies. The model was based on a static cohort of 100000 people aged ≥18 years with a monthly time-step over 40 years. The Markov model of PrEP use has eight states, ‘eligible and on PrEP’, ‘eligible but not on PrEP’, ‘ineligible and on PrEP’, ‘ineligible and not on PrEP’, ‘unidentified HIV infection on PrEP’, ‘unidentified HIV infection not on PrEP’, ‘identified HIV infection’, and ‘death’ (Supplementary Figure S1). During the 40-year simulation period, each individual had a certain probability of PrEP initiation, discontinuation, and re-initiation. According to the HIV disease progression, we divided HIV patients into 22 disease compartments [42]. The classification criteria included HIV susceptibility, HIV infection and treatment, and HIV-related deaths (Supplementary Figure S2). Further stratification and use of PrEP based on their risk of HIV infection and PrEP use (Supplementary Figure S3). After HIV infection, populations were categorized according to CD4 T-cell counts (CD4 ≥ 500, 350–499, 200–349, and <200 cells/μL). The transition probability between model states is the probability that an individual transfers from one state to another within one cycle, obtained from the published literature. We assumed all transition probabilities remained constant across the entire 40-year simulation horizon (i.e., a time-homogeneous Markov process) due to limited availability of high-quality longitudinal data on epidemiological trends and behaviour change over time among key populations. The diagnosed HIV-infected individuals would first be treated with first-line ART, and those who experienced first-line ART failure would be treated with second-line ART. We assumed that health state transition rates differed between participants on treatment and those who remained untreated. We thought that PrEP would only be offered to HIV-negative individuals from the key populations.

### National and high-incidence region scenarios

HIV incidence in China was obtained from Chinese Center for Disease Control and Prevention. High-incidence regions were defined as provinces with higher HIV incidence than the national average. Two high-incidence provinces were selected for analysis within each key population. In addition, we simulated PrEP implementation scenarios for key populations at China’s nationwide and high-incidence region levels (Table 1).

### Cost-effectiveness analysis

The health utility value is the quality-of-life adjusted weight used in the calculation of QALY, which is 1 for perfect health and 0 for death. We calculated the QALYs gained and the cost of the economic burden of HIV infection when key populations (MSM, FSW, PWID, and the HIV-negative partners of SDC) received PrEP. The analysis used QALYs as the denominator and cost monetary terms ($) as the numerator for the economic evaluation. The incremental cost-effectiveness ratio (ICER) was calculated as the difference in costs of the interventions compared with no PrEP, divided by the difference in QALYs. The willingness-to-pay (WTP) threshold was set as three times Chinese GDP per capita ($37653, 2021), drawing from WHO-CHOICE guidelines and prior literature [43, 44]. Future costs and QALYs were discounted at 3% per year [45, 46]. Benefit–cost ratio (BCR, calculated as the number of dollars saved in direct medical cost of HIV treatment for every dollar invested in PrEP and HIV testing) was also employed to evaluate the benefits of PrEP and HIV testing investment.

### Sensitivity analysis

Deterministic and probabilistic sensitivity analyses were conducted to evaluate the impact of model parameters on cost-effectiveness. One-way deterministic sensitivity analyses assessed parameter uncertainties and model robustness, while two-way sensitivity analyses examined the simultaneous variation of PrEP price and HIV incidence. Probabilistic sensitivity analysis (PSA) involved 10000 Monte Carlo simulations to describe the combined uncertainty of all model input parameters. We used preset distributions specified for the model parameters based on their own distributional patterns: gamma distributions were used for the cost parameters; the utility values and transition probabilities for each health state were distributed using a beta distribution for those whose means and standard deviations were available, and triangular distributions were used for the probability parameters for which the mean and standard deviation were unavailable because of limited data sources.

## Results

### Population impact and cost-effectiveness of PrEP among MSM in settings with various HIV incidence and PrEP price

Under no PrEP strategy nationwide, 30960 HIV infections were projected among 100000 MSM over 40 years. This would result in 2359 person-years (PYs) with AIDS and 728 HIV-related deaths. Implementing daily PrEP at 80% would reduce HIV infections to 24805 cases, 1807 PYs with AIDS, and HIV-related deaths to 563 cases, resulting in ICER $15768/QALY compared to no PrEP. Similarly, on-demand PrEP would mitigate 7636 HIV infections and 191 HIV-related deaths among 100000 MSM. PYs with AIDS would drop to 1731, translating to a 5265 QALY gain and an ICER of $4554/QALY compared to no PrEP (Table 2). The nationwide implementation of both daily and on-demand PrEP among MSM was cost-effective within the WTP threshold. Compared to daily PrEP, on-demand PrEP saved $444.07 per person and gained 0.01 QALY, with an ICER of –$44407/QALY.

**Table 2.** Results of cost-effectiveness analysis of daily and on-demand PrEP strategies compared to no PrEP for HIV prevention in China, simulation of a 100000 people cohort for four key populations

Population/region	Strategy	Total cost	PrEP and testing cost	HIV-related treatment cost	PYs on ART	HIV infections	PYs on AIDS	HIV-related deaths over the lifetime	Eff	Incr Cost	Incr Eff	ICER	BCR
**MSM**		Per capita			Per 100000				Per capita				
National	No PrEP (status quo)	2251.36	45.43	2205.93	268867	30960	2359	728	8.17	–	–	–	–
	Daily PrEP	2935.18	1223.64	1711.54	213938	24805	1807	563	8.22	683.83	0.04	15768.15	0.42
	On-demand PrEP	2491.11	903.23	1587.88	200097	23324	1731	537	8.23	239.75	0.05	4553.88	0.72
Beijing	No PrEP (status quo)	2920.85	49.35	2871.50	345598	39492	3018	930	8.12	–	–	–	–
	Daily PrEP	3445.97	1174.37	2271.60	280581	32263	2356	733	8.17	525.12	0.05	9930.80	0.53
	On-demand PrEP	2988.27	873.35	2114.92	263419	30451	2266	702	8.18	67.42	0.06	1044.53	0.92
Yunnan	No PrEP (status quo)	2417.88	46.39	2371.49	286425	32874	2508	773	8.16	–	–	–	–
	Daily PrEP	3006.53	1206.09	1800.44	223146	25784	1870	583	8.21	588.65	0.05	11737.34	0.49
	On-demand PrEP	2547.96	893.30	1654.66	206845	24041	1780	552	8.22	130.08	0.06	2129.25	0.85
**FSW**		per capita							per capita				
National	No PrEP (status quo)	48.89	32.23	16.66	2052	237	21	5	8.36	–	–	–	–
	Daily PrEP	1377.44	1365.07	12.38	1540	178	14	4	8.36	1328.55	0.00	3038298.92	0.003
	On-demand PrEP	998.94	987.47	11.47	1432	165	13	3	8.36	950.05	0.00	2606869.89	0.01
Guangxi	No PrEP (status quo)	1247.81	39.36	1208.45	148931	17105	1470	329	8.26	–	–	–	–
	Daily PrEP	2254.66	1277.33	977.33	122270	14042	1094	259	8.28	1006.84	0.02	50669.04	0.19
	On-demand PrEP	1857.31	931.42	925.89	116312	13375	1038	245	8.29	609.50	0.02	25398.82	0.32
Yunnan	No PrEP (status quo)	953.16	37.62	915.54	113373	13067	1121	251	8.29	–	–	–	–
	Daily PrEP	2035.34	1299.64	735.70	92439	10652	829	196	8.30	1082.18	0.02	70177.84	0.14
	On-demand PrEP	1641.89	945.74	696.14	87819	10133	786	186	8.31	688.72	0.02	37045.04	0.24
**PWID**		per capita							per capita				
National	No PrEP (status quo)	530.64	35.41	495.24	61838	7976	922	495	8.30	–	–	–	–
	Daily PrEP	1738.00	1321.47	416.53	52485	6721	707	398	8.31	1207.36	0.01	100988.83	0.06
	On-demand PrEP	1359.00	958.61	400.39	50555	6465	670	380	8.31	828.36	0.01	57895.49	0.1
Xinjiang	No PrEP (status quo)	2003.98	44.92	1959.07	238301	30191	3530	1888	8.11	–	–	–	–
	Daily PrEP	2882.86	1179.82	1703.04	209478	26351	2799	1573	8.15	878.87	0.04	21610.62	0.23
	On-demand PrEP	2511.81	863.94	1647.87	203199	25526	2671	1509	8.16	507.82	0.05	10361.25	0.38
Guangxi	No PrEP (status quo)	931.62	38.02	893.60	110865	14230	1650	884	8.25	–	–	–	–
	Daily PrEP	2043.31	1284.52	758.79	95064	12116	1279	719	8.27	1111.68	0.02	53732.75	0.11
	On-demand PrEP	1664.85	934.06	730.79	91757	11680	1215	687	8.27	733.23	0.02	29559.57	0.18
**SDC** ^a^		per capita							per capita				
National	No PrEP (status quo)	705.70	36.33	669.37	83993	10613	770	918	8.26	–	–	–	–
	Daily PrEP	1845.00	1320.84	524.16	67064	8494	585	715	8.29	1139.30	0.02	49701.96	0.11
	On-demand PrEP	1453.36	960.14	493.23	63450	8062	559	680	8.29	747.66	0.03	27553.34	0.19
Guangxi	No PrEP (status quo)	1323.52	40.13	1283.40	159170	19941	1453	1730	8.17	–	–	–	–
	Daily PrEP	2296.61	1276.78	1019.83	129110	16215	1120	1368	8.21	973.09	0.04	23222.30	0.21
	On-demand PrEP	1894.66	932.72	961.95	122477	15431	1073	1305	8.22	571.14	0.05	11476.15	0.36
Yunnan	No PrEP (status quo)	792.51	36.82	755.70	91450	11398	832	991	8.25	–	–	–	–
	Daily PrEP	1825.13	1306.08	519.04	63805	7931	528	600	8.29	1032.61	0.04	27615.91	0.19
	On-demand PrEP	1419.54	951.80	467.74	57791	7209	485	659	8.29	627.03	0.04	14126.08	0.31

*Note:* AIDS, acquired immunodeficiency syndrome; ART, antiretroviral therapy; BCR, benefit–cost ratio; FSW, female sex workers; ICER, incremental cost-effectiveness ratio; MSM, men who have sex with men; PWID, people who inject drugs; PYs, person-years; SDC, serodiscordant couples.

a The HIV-negative partners of SDC.

**Figure 1.**
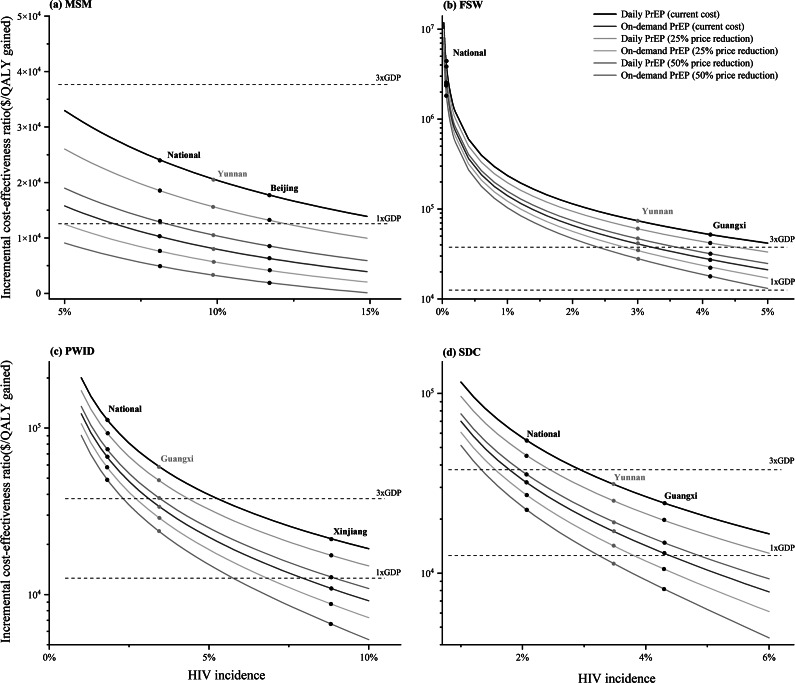
Two-way sensitivity analyses of PrEP price and HIV incidence among key populations.(a) MSM; (b) FSW; (c) PWID; (d) HIV-negative partners of serodiscordant couples (SDC).

In the two high-incidence provinces selected for MSM, Beijing (HIV incidence: 7.1/100 person-years) and Yunnan (5.3/100 person-years), compared to the national average of 4.9/100 person-years, both daily and on-demand PrEP demonstrated cost-effectiveness compared to no PrEP. In Beijing, daily PrEP had an ICER of $9931/QALY, while on demand, PrEP had $1045/QALY. In Yunnan, the corresponding ICERs were $11737/QALY for daily PrEP and $2129/QALY for on-demand PrEP (Table 2). Importantly, it was demonstrated that if the HIV incidence among MSM reached 5.0/100 PYs, both daily and on-demand PrEP would be cost-effective at a WTP threshold equivalent to three times GDP per capita ($37653) (Figure 1a).

This study presented cost-effectiveness acceptability curves across a range of WTP values, from zero to three times GDP per capita. At a threshold of three times GDP per capita, the on-demand PrEP strategy had a 100% probability of being cost-effective for the MSM population across national China (WTP = $22500), as well as in two high-incidence regions, Yunnan (WTP = $15000) and Beijing (WTP = $12500). One-way sensitivity analysis was conducted to compare the daily PrEP and on-demand PrEP strategies among MSM, the top three influential factors included the effectiveness of daily PrEP, the cost of daily PrEP per year, and the HIV incidence of MSM (Figure 3). When PrEP coverage reached 20% or 50%, on-demand PrEP retained its dominance, with ICER ranging between $4556 and $4557/QALY at national level, between $1044.5 and $1044.4/QALY in Beijing, and between $2129.8 and $2130.3/QALY in Yunnan, respectively.

### Population impact and cost-effectiveness of PrEP among FSW in settings with different HIV incidences and PrEP price

On a nationwide scale, no PrEP projected 237 HIV infections, 21 PYs with AIDS, and 5 HIV-related deaths among 100000 FSWs over the next 40 years. Compared to no PrEP, daily PrEP could lower infections to 178 cases, 14 PYs with AIDS, and HIV-related deaths to four cases, resulting in ICER $3038298/QALY; on-demand PrEP would similarly reduce 165 HIV infections, and three HIV-related deaths, and decrease to 13 PYs with AIDS per 100000 FSW, with an ICER of $2606870/QALY (Table 2). On-demand PrEP dominated daily PrEP nationally, saving $378.50 and yielding no loss in QALYs.

At the same WTP threshold, cost-effectiveness was evident when implementing on-demand PrEP exclusively for provinces (HIV incidence 1.45/100 PYs in Guangxi and 1.06/100 PYs in Yunnan vs. national HIV incidence 0.02/100 PYs) with high HIV incidences of FSW (Table 2). If HIV incidence among FSW was higher than 3.3/100 PYs, on-demand PrEP would be cost-effective within three times GDP per capita as WTP. A 25% reduction of the current price of daily PrEP would make the daily PrEP strategy cost-effective among FSWs in regions with an HIV incidence >4.5/100 PYs. (Figure 1b).

**Figure 2.**
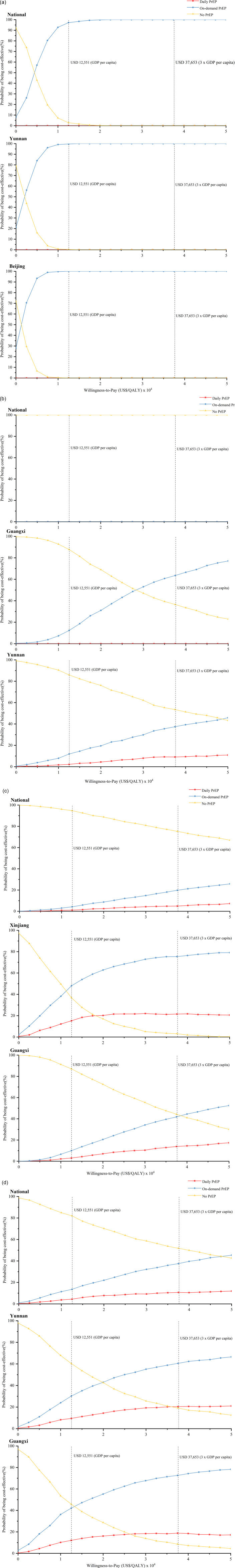
(a) Cost-effectiveness acceptability curves of MSM in different regions. (b) Cost-effectiveness acceptability curves of FSW in different regions. (c) Cost-effectiveness acceptability curves of PWID in different regions. (d) Cost-effectiveness acceptability curves of SDC in different regions. *SDC refers to the HIV-negative partners of SDC.

**Figure 3.**
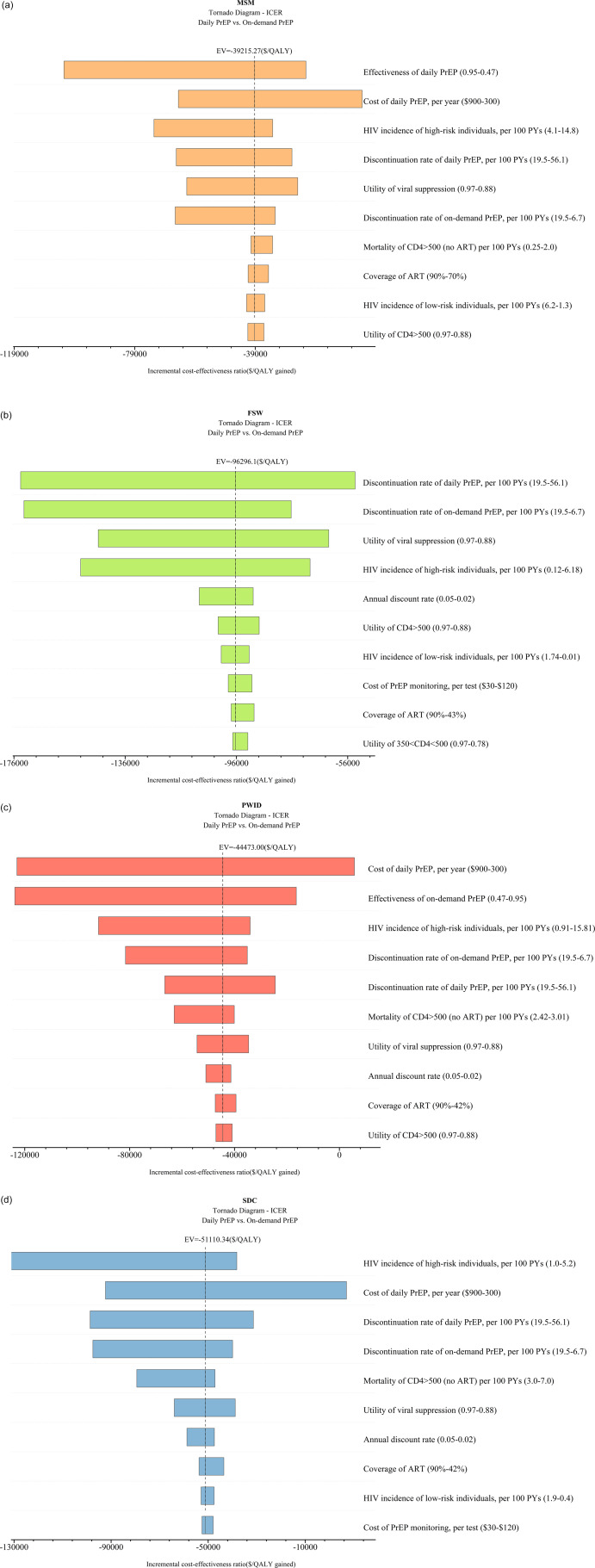
(a) Tornado diagram for Chinese MSM. (b) Tornado diagram for Chinese FSW. (c) Tornado diagram for Chinese PWID. (d) Tornado diagram for Chinese SDC. EV refers to expected values. *SDC refers to the HIV-negative partners of SDC.

For the FSW population, when the WTP was increased to three times GDP per capita, the on-demand PrEP strategy outperformed the others with a probability of being the most cost-effective at 63.5%. (Figure 2b). One-way sensitivity analysis found that the discontinuation rate of daily PrEP was the most influential factor in cost-effectiveness outcomes (Figure 3b). When PrEP coverage reached 20% or 50%, on-demand PrEP retained its dominance, with ICER ranging between $2174381 and $2176596/QALY at national level, between $25414 and $25432/QALY in Guangxi, and between $37069 and $37099/QALY in Yunnan, respectively.

### Population impact and cost-effectiveness of PrEP among PWID in settings with different HIV incidence and PrEP price

For 100000 PWID in China nationwide, no PrEP would lead to 7976 HIV infections, 922 PYs with AIDS, and 495 HIV-related deaths. Daily PrEP could mitigate infections to 6721 cases, decrease AIDS PYs to 707/100000 PWID, and lower HIV-related deaths to 398 cases compared to no PrEP. The ICER for this strategy was $100989/QALY. On-demand PrEP would further reduce infections to 6465 cases, lower AIDS PYs to 670/100000 PWID, and minimize HIV-related deaths to 380 cases compared to no PrEP. The ICER was $57895/QALY gained (Table 2). Comparing on-demand PrEP to daily PrEP, on-demand PrEP saved $379.00 per person.

In the two selected highest HIV-incidence regions of PWID in China (HIV incidence 2.77/100 PYs in Xinjiang and 1.08/100 PYs in Guangxi vs. national HIV incidence 0.57/100 PYs), the ICERs for daily PrEP were $21611/QALY in Xinjiang and $53733/QALY in Guangxi compared to no PrEP. On-demand PrEP displayed ICERs of $10361/QALY in Xinjiang and $29560/QALY in Guangxi, which showed cost-effectiveness in line with the WTP threshold set at three times GDP per capita (Table 2). Notably, if the HIV incidence among PWID reached 3.1/100 PYs, on-demand PrEP would be cost-effective, and if incidence increased to 5.4/100 PYs, daily PrEP would be cost-effective within the same WTP threshold. (Figure 1c).

No PrEP strategy for PWID had a probability of over 75% being cost-effective compared to on-demand and daily PrEP at the national level. However, in the high HIV-incidence region of PWID in Xinjiang, the on-demand PrEP strategy demonstrated superiority, with a 75.5% probability of being cost-effective when the WTP was set at three times GDP per capita. In contrast, both PrEP strategies were inferior to no PrEP in Guangxi within the WTP threshold (Figure 2c). One-way sensitivity analysis found that the cost of daily PrEP per year was the most influential factor on cost-effectiveness outcomes, followed by the effectiveness of on-demand PrEP and the HIV incidence among PWID (Figure 3c). When PrEP coverage reached 20% or 50%, on-demand PrEP retained its dominance, with ICER ranging between $57945 and $58008/QALY at national level, between $10366 and $10372/QALY in Xinjiang, and between $29582 and $29609/QALY in Guangxi, respectively.

### Population impact and cost-effectiveness of PrEP among the HIV-negative partners of SDC in settings with different HIV incidence and PrEP price

In the national scenario, among 100000 HIV-negative partners of SDC in China, no PrEP predicted 10613 HIV infections, 770 PYs with AIDS, and 918 HIV-related deaths over 40 years. Implementing daily PrEP could lower infections to 8494 cases, decrease to 585 PYs with AIDS per 100000 HIV-negative partners of SDC, and reduce HIV-related deaths to 715 cases. The ICER for this strategy is $49702/QALY compared to no PrEP. On-demand PrEP would mitigate 8062 HIV infections and 680 HIV-related deaths and decrease AIDS to 559/100000 HIV-negative partners of SDC compared to no PrEP. The ICER is $27553/QALY gained, which was cost-effective at the WTP threshold (Table 2). Comparing on-demand PrEP to daily PrEP, on-demand PrEP dominated daily PrEP, saving $391.64 per person.

Compared to no PrEP, in the two provinces with the highest HIV incidence regions for the HIV-negative partners of SDC in China (HIV incidence 2.50/100 PYs in Guangxi and 1.40/100 PYs in Yunnan vs. national HIV incidence 1.20/100 PYs), the ICERs for daily PrEP were $23222/QALY in Guangxi and $27616/QALY in Yunnan. Similarly, on-demand PrEP displayed ICERs of $11476/QALY in Guangxi and $14126/QALY in Yunnan under the WTP threshold (Table 1). Importantly, if the HIV incidence among the HIV-negative partners of SDC reached 1.5/100 PYs, on-demand PrEP would be cost-effective, and if incidence increased to 1.9/100 PYs, daily PrEP would be cost-effective within the same WTP threshold (Figure 1d).

According to the cost-effectiveness acceptability curves, in Guangxi, a high-incidence region, on-demand PrEP strategy outperformed both the daily PrEP and no PrEP strategies among HIV-negative partners of the SDC when the WTP threshold was standardized to 1.1 times the GDP per capita ($13806) (Figure 2d). ICER was notably influenced by the HIV incidence of PWID, cost of daily PrEP per year, and discontinuation rate of daily PrEP per 100 PYs (Figure 3d). When PrEP coverage reached 20% or 50%, on-demand PrEP retained its dominance, with ICER ranging between $27575 and $27602/QALY at national level, between $11483 and $11492/QALY in Guangxi, and between $14137 and $14151/QALY in Yunnan, respectively.

## Discussion

The study investigates the cost-effectiveness of implementing PrEP among key populations in China, revealing that the optimal strategy is to implement on-demand PrEP for preventing HIV transmission among MSM and the HIV-negative partners of SDC nationwide while targeting high-incidence regions for FSW and PWID. Compared to no PrEP, the implementation of on-demand PrEP is projected to yield gains in QALYs and avert approximately 20% of new HIV infections over 40 years of 100000 individuals within all four key populations in China. Moreover, our analysis indicates that on-demand PrEP consistently dominates daily PrEP across all populations and scenarios, providing comparable or greater health benefits at substantially lower cost. Our findings underscore the viability of integrating PrEP into China’s national HIV prevention programme, prioritizing its application among the HIV-negative partners of SDC and MSM populations initially, and among pilot regions with high HIV incidence for FSW and PWID.

Our model indicates that both daily PrEP and on-demand PrEP among MSM are highly cost-effective and can avert the largest proportion of new HIV infections. These findings are consistent with other research, suggesting that implementing PrEP strategies would be cost-effective [47], and can reduce HIV incidence among MSM [48]. Despite an increase in awareness of PrEP among MSM over the years, a significant gap persists between the objective need for PrEP, willingness to use it, affordability, and actual uptake [49]. Considering large population and the social and economic implications in China, our findings emphasize the urgent need to implement PrEP nationwide among MSM. This approach enables early prevention and control measures, thereby minimizing potential losses.

In HIV-negative partners of SDC, our study finds that only on-demand PrEP is cost-effective compared to no PrEP, while daily PrEP is not. Furthermore, when directly comparing the two strategies, on-demand PrEP dominates daily PrEP by achieving similar or greater health benefits at a significantly lower cost. These findings differ from some studies [26, 28, 50], which showed that daily PrEP can be cost-effective. The difference may be attributed to multiple factors, such as additional policy interventions, the viral suppression status of HIV-positive partners, the frequency of sexual activity, and the use of preventive measures. In China, the U = U (Undetectable equals Untransmittable) policy is applied, leading to nearly 100% ART coverage for HIV-positive partners of SDC, which may reduce transmission risks and diminish the additional preventive benefits of daily PrEP [51]. Moreover, on-demand PrEP reduces overall drug usage and improves adherence, contributing to its overall cost-effectiveness. Although our study finds that on-demand PrEP appears to be the only cost-effective strategy in HIV-negative partners of SDC, further research is needed to explore how real-world adherence, long-term clinical outcomes, and regional differences in implementation affect cost-effectiveness.

While PrEP does not show significant preventive effects among FSW and PWID at the national level, our model indicates that in high-incidence regions, on-demand PrEP is cost-effective for all key populations. The lower cost-effectiveness of PrEP in the FSW and PWID may be attributed to the relatively low HIV incidence in China. According to WHO guidelines, PrEP is recommended for populations with HIV incidence at or greater than 3 cases per 100PYs [52]. Nevertheless, a study projecting the impact and cost-effectiveness of PrEP interventions across 13 low-resource countries suggested that combined interventions could prove more effective for FSWs [28]. In high-incidence regions, PrEP might be recommended, especially in cases where condom use may not provide sufficient protection [53]. Notably, the effectiveness of PrEP among PWID appears to be lower compared to the MSM population primarily due to challenges related to awareness and adherence [54, 55]. Therefore, future efforts should focus on developing and implementing strategies that improve adherence and awareness of PrEP among PWID, ensuring that the approach addresses the specific needs and circumstances of this population. Existing evidence suggests that combining PrEP with methadone maintenance treatment (MMT) could be a more effective strategy, particularly as the prevalence of heroin use decreases [56, 57]. Hence, it is imperative to implement combined interventions among key populations, including FSWs and PWID, to gain crucial insights into the feasibility and optimal strategies for integrating PrEP into national HIV prevention programmes. These strategies require careful design, taking into account the unique characteristics, risk profiles, and socio-cultural factors of each population, ensuring that PrEP recommendations align with their specific needs and epidemiological context. Another issue to consider is how to achieve 80% PrEP coverage among key populations. Due to marginalization, access to facility-based PrEP services remains suboptimal. Therefore, in real-world settings, community-based delivery of services, such as safe HIV testing, PrEP provision, and linkage to care, may be more effective [58].

Based on nationwide and high-incidence region simulations, on-demand PrEP is a more cost-effective option than daily PrEP among all key populations, particularly considering its current price in China. Lowering the cost of PrEP would lead to more favourable ICERs, making the strategy more cost-effective. Previous research has suggested that on-demand PrEP reduces the ICER to a cost-effective range, and a daily generic regimen based on tenofovir offers even greater cost-effectiveness [11]. However, it should be acknowledged that the current price of PrEP is a significant barrier to uptake, especially when compounded by its exclusion from health insurance coverage. Given that on-demand PrEP has demonstrated a lower cost for preventing new HIV infections, not only China but other low- and middle-income countries (LMICs) may find on-demand PrEP to be an optimal strategy compared to daily PrEP, which may be potentially more cost-effective [59]. Moreover, the cost of PrEP may also introduce a barrier to optimal PrEP adherence and discontinuation [60]. Previous studies have found that adherence to on-demand PrEP was higher than daily PrEP, and adherence to daily PrEP tended to decrease over time [61], increasing the risk of HIV infections and drug resistance [62]. In this context, the introduction of long-acting injectable PrEP may provide a solution by addressing adherence barriers, reducing the burden of frequent dosing, and potentially improving cost-effectiveness in the long term [63]. Further comparative analyses are warranted to explore the economic viability of these emerging PrEP options in China. To improve accessibility to PrEP and enhance its overall cost-effectiveness, we strongly recommend that the Chinese government consider measures such as reducing the price of PrEP and integrating it into the national health insurance system.

Notably, differences in cost-effectiveness findings across studies may also stem from variations in the WTP thresholds applied. While some studies adopted absolute national WTP thresholds (e.g., country-specific monetary values), others used relative measures, such as one to three times GDP per capita, as recommended by the WHO. However, the lack of explicit WTP specification in some publications limits direct comparability. In our study, we adopted a standardized approach by using a WTP threshold equivalent to three times China’s per capita GDP, in line with WHO guidelines, to ensure greater comparability and policy relevance. Furthermore, we employed cost-effectiveness acceptability curves to present the probability of cost-effectiveness across a full range of WTP values (from 0 to 3 × GDP per capita), thus accommodating variations in policy preferences or thresholds across settings. This approach helps mitigate interpretation bias arising from arbitrary or inconsistent WTP thresholds used in different studies.

Several limitations should be noted. First, the parameters estimated from diverse sources introduce variability in data quality. In the absence of region-specific, age-disaggregated HIV incidence and mortality data, we used estimates for adults aged 18 and above, assuming an average age at model entry. In particular, the lack of parameter estimates specific to FSW required us to adopt values from the broader female population. These factors may contribute to uncertainty in our findings, although we conducted a PSA with 10000 Monte Carlo simulations to account for the combined uncertainty of all input parameters. Second, the model assumed a constant HIV incidence rate and fixed transition probabilities over the 40-year projection period. This assumption does not capture the evolving HIV prevention and treatment landscape in China, as the impact of future interventions remains uncertain. Emerging strategies, such as U=U (undetectable = untransmittable), long-acting injectable PrEP, and test-and-treat initiatives, are expected to reduce HIV incidence over time, and the model may overestimate the long-term benefits and cost-effectiveness of PrEP. Third, the use of the healthcare system perspective rather than a broader societal perspective represents a limitation. While this approach is consistent with current pharmacoeconomic guidelines in China, it may overlook important indirect costs and benefits. Future research could incorporate these factors using a societal perspective, possibly by integrating productivity loss estimates, patient time costs, and broader public health externalities into the model. Finally, our study did not include formal model calibration or validation against real-world HIV surveillance data primarily due to the limited availability of high-quality, disaggregated, and PrEP-stratified incidence data in China. Future studies may benefit from dynamic transmission modelling approaches that can be more readily calibrated to empirical cohort data for key populations. Despite these limitations, this study represents the first cost-effectiveness investigation exploring both HIV daily PrEP and on-demand PrEP for MSM, FSW, PWID, and the HIV-negative partners of SDC populations in China. In addition, as we adopted a wide range of parameters for various key populations in our model, the uncertainty of parameters like uptake, adherence, and cost of PrEP would have a great impact on the cost-effectiveness of strategies. However, these sensitivities can also inform how policymakers implement PrEP strategies in different regions, with different HIV disease burdens, and in different key populations to make the strategies cost-effective in certain contexts.

## Conclusion

This study indicates that both daily PrEP and on-demand PrEP effectively reduce new HIV infections among MSM, while only on-demand PrEP shows significant preventive effects for HIV-negative partners of SDC. In high HIV-incidence regions, on-demand PrEP shows substantial effectiveness in mitigating HIV incidence among FSW and PWID. Moreover, on-demand PrEP is a more cost-effective strategy than daily PrEP in key populations, making it a viable strategy for nationwide implementation. However, the cost-effectiveness might be hindered by the cost of the PrEP, acceptance, and adherence, which need to be fully considered in policy development and promotion. Therefore, it is recommended that on-demand PrEP be prioritized as a promotion strategy, especially for HIV-negative partners of SDC and in high-incidence regions. The insights from this study provide critical evidence for shaping HIV prevention and control policies in China while also contributing valuably to the global endeavours aimed at achieving the ambitious goal of HIV elimination by 2030.

## Supporting information

10.1017/S095026882510068X.sm001 Su et al. supplementary material 1 Su et al. supplementary material

10.1017/S095026882510068X.sm002 Su et al. supplementary material 2 Su et al. supplementary material

## Data Availability

The data supporting this study’s findings are available in this article’s supplementary material.

## Supplementary material

The supplementary material for this article can be found at http://doi.org/10.1017/S095026882510068X.
