# Supplementary material for: Epidemiological impacts and cost-effectiveness of daily and on-demand oral pre-exposure prophylaxis among key HIV populations in China: An economic evaluation
Source: Epidemiol Infect. 2025 Dec 19;153:e139. doi: 10.1017/S095026882510068X (PMC12722553; doi:10.1017/S095026882510068X)
Supplement: Su et al. supplementary material 2 — Su et al. supplementary material [file S095026882510068Xsup002.docx]

**Supplementary materials**

**
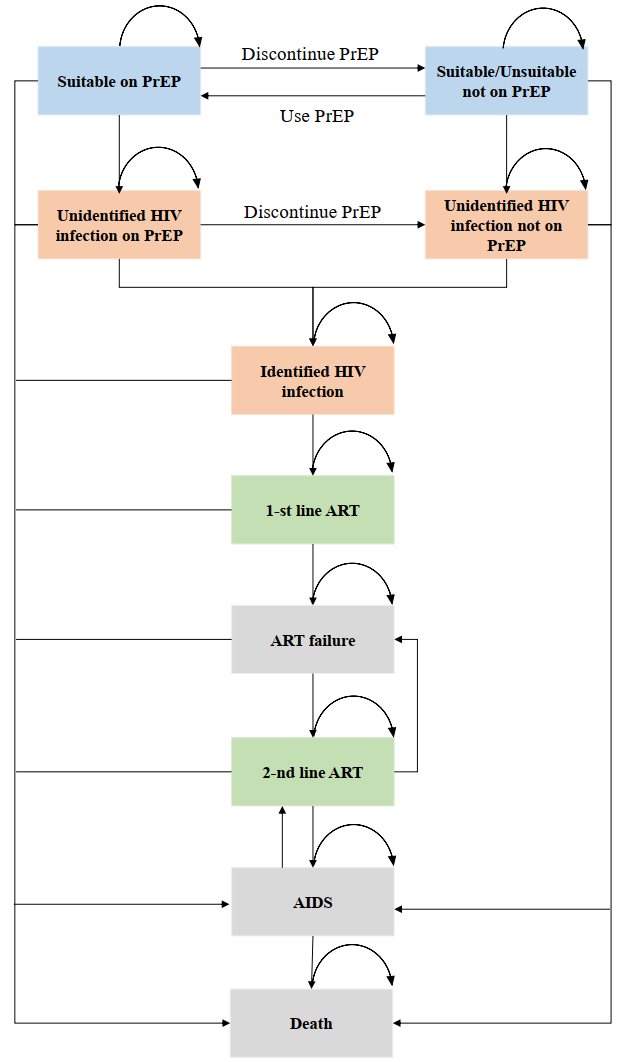
**

**Appendix 1: Figure S1 Markov Model structure**


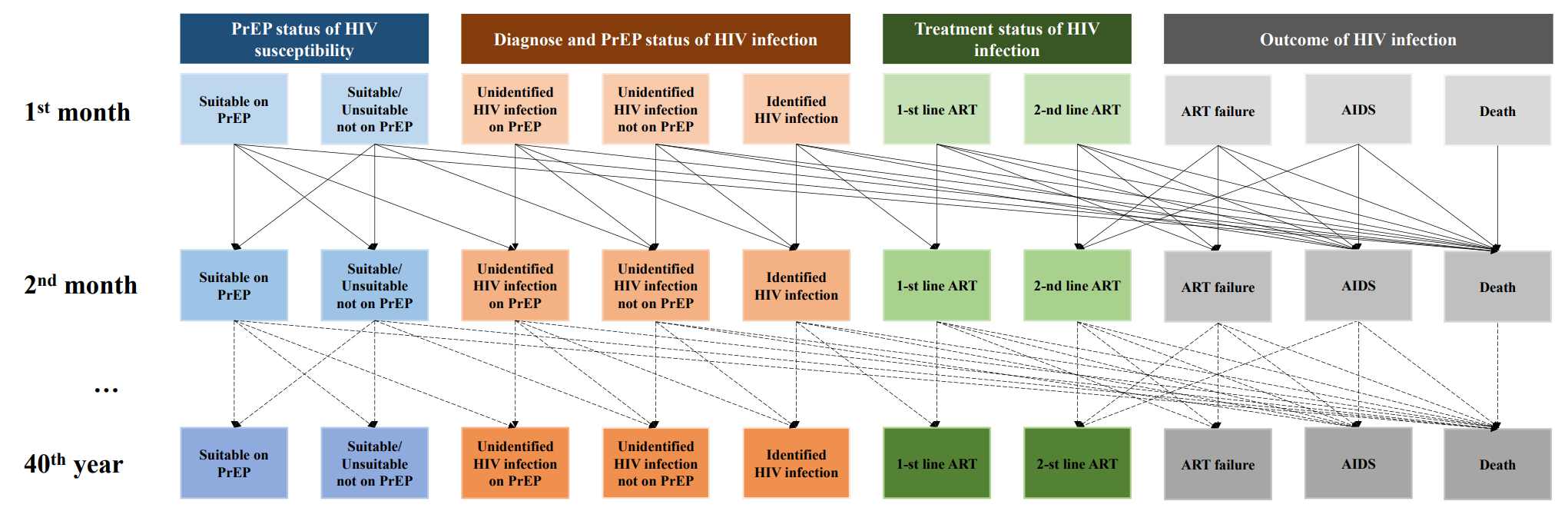


**Figure S2 Transitions between different Markov states of HIV infection, diagnosis, and treatment**

Rectangles represent health states, and lines with arrows represent transitions and their directions. Model cycle length was 1 month and over a 40 year period (estimated expected life span of HIV-positive adults among four key populations). Each Markov state can be transferred to another state according to a certain transfer probability. The model can be divided into four parts, HIV susceptible state, HIV infected state, treated state and progressed state after HIV infection. Those who survived and remain HIV-negative were categorized into two states: sutiable and on PrEP and sutiable/unsutiable but not on PrEP. Once MSM, FSW, PWID, and the HIV-negative partners of SDC acquire HIV, they would start out in the undiagnosed state with CD4 cell levels >500, and then after diagnosed, they moved into the identified HIV infection status. The individuals who identified HIV infection would first receive first-line ART according to the treatment coverage of the different key populations, and those who failed first-line ART would receive second-line ART. Those who discontinue ART or experience ART failure will continue to progress according to the natural history of HIV disease. AIDS can only be switched to second-line ART if ART is restarted. All transition probabilities between Markov states (e.g., HIV acquisition, diagnosis, ART initiation, treatment failure) were assumed to be constant over time and did not vary by month or year.

**Figure S3 Transitions between Markov states of suitable on PrEP, suitable not on PrEP, and unsuitable not on PrEP**

(Circles with "M" represent Markov states; gray circles represent "Death", i.e., absorbing states; rectangles represent transition states)

**Table S1 List of parameters used in Chinese HIV key populations**

| **Parameter** | **Baseline(range)** | **Distribution** | **Reference** |
| --- | --- | --- | --- |
| ***HIV incidence (/100 person years)*** |  |  |  |
| National |  |  | [1-3] |
| MSM | 4.93 (4.15-5.72) | Beta (23.06, 444.64) |  |
| FSW | 0.02 (0.01-0.04) | Beta (3.99, 19991.00) |  |
| PWID | 0.57 (0.43-0.72) | Beta (32.30, 5634.21) |  |
| SDC* | 1.2 (0.9-1.7) | Beta (1.41, 116.15) |  |
| Beijing |  |  | [4] |
| MSM | 7.1 (6.4-7.7) | Beta (46.76, 611.83) |  |
| Guangxi |  |  | [5-7] |
| FSW | 1.45 (1.17-1.76) | Beta (2.06, 139.84) |  |
| PWID | 1.08 (0.38-1.79) | Beta (1.14, 104.69) |  |
| SDC* | 2.5 (2.2-2.7) | Beta (6.07, 236.68) |  |
| Yunnan |  |  | [8-10] |
| MSM | 5.32 (2.66-7.98) | Beta (26.74, 475.95) |  |
| FSW | 1.06 (0.74-1.47) | Beta (1.10, 102.78) |  |
| SDC* | 1.4 (0.7-2.1) | Beta (1.92, 135.12) |  |
| Xinjiang |  |  | [11] |
| PWID | 2.77 (2.15-3.52) | Beta (7.43, 260.89) |  |
| ***Mortality, per 100 PYs*** |  |  |  |
| CD4 <200 on ART |  |  | [12-15] |
| MSM | 9.0 (7.5-22.0) | Beta (73.62, 744.38) |  |
| FSW | 3.4 (1.7-5.1) | Beta (11.13, 316.31) |  |
| PWID | ﻿6.68 (6.02-7.34) | Beta (43.03, 589.73) |  |
| SDC* | 5 (2.5-7.5) | Beta (23.70, 450.30) |  |
| CD4 <200 no ART |  |  | [11-13, 16] |
| MSM | 39.0 (9.0-53.7) | Beta (8.89, 13.90) |  |
| FSW | 2.8 (2.71-2.9) | Beta (7.59, 263.57) |  |
| PWID | 14.83 (12.79-16.88) | Beta (1.72, 9.91) |  |
| SDC* | 25 (20-30) | Beta (4.44, 13.31) |  |
| 200<CD4 <350 on ART |  |  | [12-15] |
| MSM | 1.54 (0.56-5.0) | Beta (2.32, 148.31) |  |
| FSW | 3.4 (1.7-5.1) | Beta (11.13, 316.31) |  |
| PWID | ﻿3.87 (3.24-4.51) | Beta (14.36, 356.66) |  |
| SDC | 1 (0.5-1.5) | Beta (0.98, 97.02) |  |
| 200<CD4 <350 no ART |  |  | [11-13, 16] |
| MSM | 6.0 (3.0-9.0) | Beta (33.78, 529.22) |  |
| FSW | 2.8 (2.71-2.9) | Beta (7.59, 263.57) |  |
| PWID | 5.99 (5.13-6.85) | Beta (33.67, 528.45) |  |
| SDC* | 15 (10-20) | Beta (1.76, 9.99) |  |
| 350<CD4<500 on ART |  |  | [12-15] |
| MSM | 1.0 (0.56-5.0) | Beta (0.98, 97.02) |  |
| FSW | 3.4 (1.7-5.1) | Beta (11.13, 316.31) |  |
| PWID | ﻿2.33 (1.57-3.08) | Beta (5.28, 221.29) |  |
| SDC* | 1 (0.5-1.5) | Beta (0.98, 97.02) |  |
| 350<CD4<500 no ART |  |  | [11-13, 16] |
| MSM | 2.0 (1.0-6.3) | Beta (3.90, 191.10) |  |
| FSW | 0.85 (0.67-1.03) | Beta (71.63, 8355.12) |  |
| PWID | 2.71 (2.42-3.01) | Beta (7.12, 255.54) |  |
| SDC* | 5 (3-7) | Beta (23.70, 450.30) |  |
| CD4>500 on ART |  |  | [12-15] |
| MSM | 0.5 (0.25-2.0) | Beta (24.87, 4949.13) |  |
| FSW | 3.4 (1.7-5.1) | Beta (11.13, 316.31) |  |
| PWID | ﻿2.33 (1.57-3.08) | Beta (5.28, 221.29) |  |
| SDC* | 1 (0.5-1.5) | Beta (0.98, 97.02) |  |
| CD4>500 no ART |  |  | [11-13, 16] |
| MSM | 0.5 (0.25-2.0) | Beta (24.87, 4949.13) |  |
| FSW | 1.08 (0.73-1.43) | Beta (1.14, 104.69) |  |
| PWID | 2.71 (2.42-3.01) | Beta (7.12, 255.54) |  |
| SDC* | 5 (3-7) | Beta (23.70, 450.30) |  |
| **Discontinuation rate on ART** |  |  |  |
| CD4<200 |  |  | [17, 18] |
| MSM, FSW | 0.069 (0.0345-0.1035) | Beta (44.26, 597.13) |  |
| PWID | 0.165 (0.0825-0.2475) | Beta (2.11, 10.67) |  |
| SDC* | 0.072 (0.031-0.108) | Beta (48.04, 619.12) |  |
| 200<CD4<350 |  |  | [17, 18] |
| MSM, FSW | 0.041 (0.0205-0.0615) | Beta (16.08, 376.11) |  |
| PWID | 0.165 (0.0825-0.2475) | Beta (2.11, 10.67) |  |
| SDC* | 0.072 (0.031-0.108) | Beta (48.04, 619.12) |  |
| 350<CD4<500 |  |  | [17, 18] |
| MSM, FSW | 0.045 (0.0225-0.0675) | Beta (19.29, 409.46) |  |
| PWID | 0.165 (0.0825-0.2475) | Beta (2.11, 10.67) |  |
| SDC* | 0.072 (0.031-0.108) | Beta (48.04, 619.12) |  |
| CD4<500 |  |  | [17, 18] |
| MSM, FSW | 0.045 (0.0225-0.0675) | Beta (19.29, 409.46) |  |
| PWID | 0.165 (0.0825-0.2475) | Beta (2.11, 10.67) |  |
| SDC* | 0.072 (0.031-0.108) | Beta (48.04, 619.12) |  |
| ***HIV/AIDS treatment*** |  |  |  |
| ART Coverage |  |  | [19] |
| MSM | 0.83 (0.7-0.9) | Beta (7.99, 1.95) |  |
| FSW | 0.86 (0.43-1) | Beta (9.49, 1.55) |  |
| PWID | 0.83 (0.415-1) | Beta (7.99, 1.95) |  |
| SDC* | 0.8528 (0.4264-1) | Beta (9.99, 1.76) |  |
| 1-st line ART failure rate |  |  | [20, 21] |
| MSM, FSW, PWID, SDC* | 0.071 (0.065-0.176) | Beta (46.76, 611.83) |  |
| 2nd/3rd line ART failure rate |  |  | [22] |
| MSM, FSW, PWID, SDC* | 0.088 (0.071-0.109) | Beta (70.54, 731.02) |  |
| ***Effectiveness of daily PrEP, RR*** |  |  | [23-25] |
| MSM, FSW | 0.472 (0.141-0.95) | Beta (301.76, 66.24) |  |
| PWID | 0.49 (0.096-0.70) | Beta (11.76, 12.23) |  |
| SDC* | 0.75 (0.375-1) | Beta (13.31, 4.44) |  |

*SDC refers to the HIV-negative partners of SDC

**References**

1. Zhang L, Chow EP, Jing J, Zhuang X, Li X, He M, et al. HIV prevalence in China: integration of surveillance data and a systematic review. Lancet Infect Dis. 2013 Nov;13(11):955-63.

2. Wang L, Peng Z, Li L, Norris JL, Wang L, Cao W, et al. HIV seroconversion and prevalence rates in heterosexual discordant couples in China: a systematic review and meta-analysis. AIDS Care. 2012;24(9):1059-70.

3. He J, Ju H, Wu C. [Incidence of new HIV infection and its influencing factors among men who have sex with men in China: a meta-analysis.]. Preventive Medicine. 2022;34(1):70-7.

4. Jia Z, Huang X, Wu H, Zhang T, Li N, Ding P, et al. HIV burden in men who have sex with men: a prospective cohort study 2007-2012. Sci Rep. 2015 Jul 2;5:11205.

5. Chen J, Liao JY, Nong HT. [Analysis of new HIV infections and its influencing factors among drug users in Guixi region, 2013-2015.] Modern Preventive Medicine 2018;45(17):3207-11.

6. Song QY, Yang XY, Jiang H, jgh. [Preventive effect of antiretroviral therapy on the positive transfer of antibodies in HIV-negative spouses of mono-positive families in Guangxi Zhuang Autonomous Region.]. Chinese Journal of Epidemiology. 2015;36(12):1401-5.

7. Li J, Zhang H, Shen Z, Zhou Y, Fang N, Wang L, et al. Screening for Acute HIV Infections and Estimating HIV Incidence among Female Sex Workers from Low-Grade Venues in Guangxi, China. PLOS ONE. 2014;9(6):e99522.

8. Zhu P. [AIDS cohort study of HIV-infected spouses.]: Fudan University; 2012.

9. Ma J, Li YF, Zhang RZ, dyd. [A cohort study of new HIV infections among MSM in Kunming, 2012-2016.]. Chinese Journal of AIDS & STD. 2017;23(08):755-7+63.

10. Su Y. [Epidemiologic study of AIDS among clandestine prostitutes and clients of prostitutes in Kaiyuan City, Yunnan Province, China.]: Chinese Center for Disease Control and Prevention; 2015.

11. Zhou J, Ni YK, Chen XL, Ma YY HX, Ni MJ. [Analysis of the rate of new HIV infections and its influencing factors among intravenous drug users in Xinjiang.]. Chinese Journal of Public Health 2018;34(12):1587-91.

12. Liu E, Rou K, McGoogan JM, Pang L, Cao X, Wang C, et al. Factors associated with mortality of HIV-positive clients receiving methadone maintenance treatment in China. J Infect Dis. 2013;208(3):442-53.

13. Ma L. [Study the cost, effect, and utility of different antiretroviral treatment strategies for HIV single-positive families.] Chinese Center for Disease Control and Prevention 2016.

14. Cai C, Tang HL, Li DM, F L. [Analysis of mortality trend and its related risk factors of AIDS patients in China.] Chinese Medicla Association Journals. 2021;42(01):121-5.

15. Zhong Y. Economic Evaluation of PrEP Prevention Strategies in MSM Population: Chongqing Medical University; 2018.

16. Tang Z, Pan SW, Ruan Y, de Bekker-Grob E. Effects of high CD4 cell counts on death and attrition among HIV patients receiving antiretroviral treatment: an observational cohort study. Sci Rep. 2017;7(1):3129.

17. Jiamsakul A KS, Ng OT, et al. Long-term loss to follow-up in the TREAT Asia HIV Observational Database (TAHOD). HIV Med. 2019;20(7):439-49.

18. Mai L. [Shedding rate and survival analysis of HIV/AIDS patients receiving ART in China.]: Peking Union Medical College; 2016.

19. UNAIDS. UNAIDS Data 2019. 2019.

20. Wu Z. [AIDS prevention and treatment strategies with Chinese characteristics.]. Chinese Journal of Disease Control 2019;23(08):885-9.

21. Ma W. [Epidemiologic evaluation of the effectiveness of antiretroviral treatment for AIDS in some areas of China.]: Nanjing Medical University; 2016.

22. Boettiger DC, Nguyen VK, Durier N, Bui HV, Heng Sim BL, Azwa I, et al. Efficacy of second-line antiretroviral therapy among people living with HIV/AIDS in Asia: results from the TREAT Asia HIV observational database. J Acquir Immune Defic Syndr. 2015 Feb 1;68(2):186-95.

23. Choopanya K, Martin M, Suntharasamai P, Sangkum U, Mock PA, Leethochawalit M, et al. Antiretroviral prophylaxis for HIV infection in injecting drug users in Bangkok, Thailand (the Bangkok Tenofovir Study): a randomised, double-blind, placebo-controlled phase 3 trial. Lancet. 2013 Jun 15;381(9883):2083-90.

24. Shen Y, Lu H. [Application and Challenges of Pre-Exposure Prophylaxis for Human Immunodeficiency Virus.]. Journal of Zhejiang University (Medical) 2016;45(03):221-7.

25. Wu D, Tao H, Dai J, Liang H, Huang A, Zhong X. Study on Pre-Exposure Prophylaxis Regimens among Men Who Have Sex with Men: A Prospective Cohort Study. Int J Environ Res Public Health. 2019 Dec 9;16(24).
